# Ongoing Coevolution of *Wolbachia* and a Widespread Invasive Ant, *Anoplolepis gracilipes*

**DOI:** 10.3390/microorganisms8101569

**Published:** 2020-10-12

**Authors:** Chih-Chi Lee, Chun-Yi Lin, Shu-Ping Tseng, Kenji Matsuura, Chin-Cheng Scotty Yang

**Affiliations:** 1 Laboratory of Insect Ecology, Graduate School of Agriculture, Kyoto University, Kyoto 606-8502, Japan; lee.chihchi.54m@st.kyoto-u.ac.jp; 2 Research Institute for Sustainable Humanosphere, Kyoto University, Kyoto 611-0011, Japan; chunyitonylin@gmail.com; 3 Department of Entomology, University of California, 900 University Avenue, Riverside, CA 92521, USA; magic760812@gmail.com; 4 Department of Entomology, Virginia Polytechnic Institute and State University, Blacksburg, VA 24061, USA; 5 Department of Entomology, National Chung Hsing University, Taichung 402204, Taiwan

**Keywords:** ddRAD sequencing, invasive species, mitochondrial DNA, population genomics, *Wolbachia*, yellow crazy ant

## Abstract

While *Wolbachia* are commonly found among arthropods, intraspecific infection rates can vary substantially across the geographic populations. Here we report nearly 100% prevalence of *Wolbachia* in the global populations of the yellow crazy ant, *Anoplolepis gracilipes*. To understand coevolutionary history between *Wolbachia* and *A. gracilipes*, we identified single nucleotide polymorphisms (SNPs) in *Wolbachia* from the ant across 12 geographical regions and compared the phylogeny of SNP-based *Wolbachia* to patterns of the ant’s mitochondrial DNA (mtDNA) variation. Our results revealed a strong concordance between phylogenies of *Wolbachia* and host mtDNA, providing immediate evidence of co-divergence. Among eight identified SNP loci separating the genetic clusters of *Wolbachia*, seven loci are located in potential protein-coding genes, three of which being non-synonymous SNPs that may influence gene functions. We found a *Wolbachia* hypothetical protein gene with signature of positive selection. These findings jointly allow us to characterize *Wolbachia*-ant coevolution and also raise a question about mechanism(s) underlying maintenance of high prevalence of *Wolbachia* during the colonization of this invasive ant.

## 1. Introduction

*Wolbachia* is an endosymbiont commonly found in arthropods and filarial nematodes [1]. This bacterium could manipulate the reproduction of its arthropod hosts to benefit itself as a reproductive parasite. The reproductive manipulations include cytoplasmic incompatibility, male killing, feminization, and induction of parthenogenesis [1,2]. Evidence has been accumulated recently in suggesting that *Wolbachia* confers benefits to their hosts and acts as a mutualistic partner that is involved in either nutritional provisioning or host pathogen defense [2]. In more extreme cases *Wolbachia* can be an obligate mutualist with fitness costs otherwise being significant when *Wolbachia* is removed/cured from the hosts [2,3]. Such intimate interactions between the symbiont and host would lead to coevolution and high infection rate of *Wolbachia* (e.g., 90–100%) within the host population. For example, the coevolutionary pattern (e.g., co-divergence or co-speciation) is commonly observed in filarial nematodes [4] and bedbugs [5] as *Wolbachia* functions as a nutritional mutualist [5,6] and often persists within and among species at 100% prevalence [5,7]. While infections up to 100% have been occasionally reported in *Wolbachia* acting as a reproductive parasite in arthropods [8,9], a high level of *Wolbachia* prevalence within arthropod populations seems uncommon [10,11]. The role of *Wolbachia* in ants, however, is relatively under-described and yet likely varies across species. Most studies have found limited evidence supporting the existence of *Wolbachia*-induced cytoplasmic incompatibility, parthenogenesis, and feminization as viable reproductive manipulation in ant [12] (but see a recent study [13] for evidence of cytoplasmic incompatibility).

The yellow crazy ant, *Anoplolepis gracilipes*, is a widespread invasive ant that has posed significant threats to local biodiversity and ecosystem sustainability in most of its introduced range [14]. This ant is polygynous (nest headed by multiple queens), polydomous (a colony inhabiting in multiple spatially separated yet socially connected nests), and often forms supercolonies (a supercolony comprises of physically separated nests that are mutually tolerant of each other). Like many invasive ant species, independent foundation (e.g., nuptial flight) is rare in *A. gracilipes*, and the colony reproduction is primarily through dependent foundation (e.g., budding) [15,16]. The limited female dispersal ability and potential intranidal mating have been shown to restrict gene flow among different nests of *A*. *gracilipes* even within the same supercolony and thus contribute to within-supercolony divergence [17]. Consequently, these life-history traits may also facilitate divergence of *Wolbachia* in the ant, especially given the fact that this endosymbiont is maternally transmitted.

Both vertical and horizontal transmissions were reported in *Wolbachia* [1]. Previous studies and our preliminary data have shown that *A*. *gracilipes* collected from several Indo-Pacific islands and Australia including Christmas Island share an identical *Wolbachia wsp* genotype [18,19], and that the bacterium seems to persist at high prevalence across these populations [18,19]. Coupled with a recent study that revealed no evidence of horizontal transfer of *Wolbachia* between *A. gracilipes* and its closely associated kleptoparasitic ant crickets [18], it is likely that *Wolbachia* may have been primarily transferred vertically in *A. gracilipes*, thus providing an excellent opportunity to explore *Wolbachia*–host coevolutionary history.

In the present study, we conducted survey of *Wolbachia* prevalence and infection status (single or multiple strains infections) in *A*. *gracilipes* at a global scale (a total of 12 geographic regions including Southeast and East Asia, Sri Lanka, several Pacific islands, and Australia). We also assessed and compared phylogenies of *Wolbachia* (based on genomic single nucleotide polymorphisms (SNPs)) and partial sequence of mitochondrial DNA of *A*. *gracilipes* to examine whether the coevolution between *Wolbachia* and the ant holds true. Lastly, we tested whether there is signature of natural selection in *Wolbachia* to reflect potential *Wolbachia*–ant coevolutionary history.

## 2. Materials and Methods

### 2.1. Sample Collection, DNA Extraction and Molecular Genetic Assays

**Figure 1:**
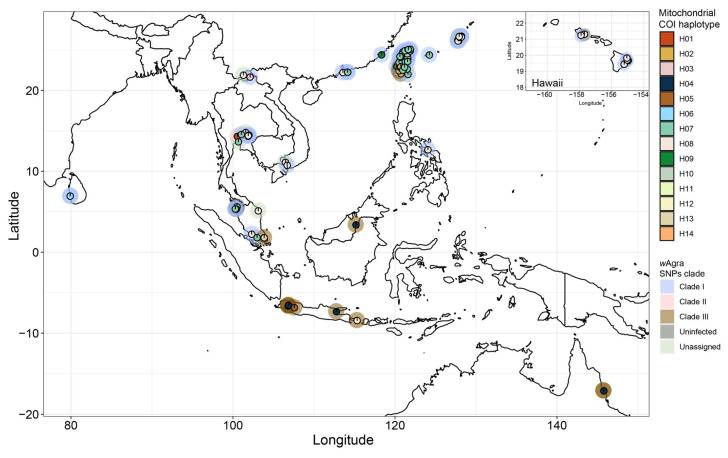
Geographical distribution, mitochondrial *COI* haplotype (H01-H14, inner circle) and *w*Agra SNPs clade (outer circle) of all *A*. *gracilipes* colonies in this study.

*Wolbachia* prevalence was assessed based on 80 yellow crazy ant colonies from a total of 12 geographical regions including Southeast and East Asia, Sri Lanka, several Pacific islands, and Australia collected from 2012 to 2019 (Figure 1, Supplementary Materials Table S1). The collected specimens were preserved in 95% ethanol upon DNA extraction. To examine *Wolbachia* infection and identify the respective *Wolbachia* strain, three random adult worker ants per ant colony were selected. The whole genomic DNA was purified individually from the whole ant body using QIAamp DNA Mini Kit (Qiagen, Valencia, CA, USA) following the manufacture’s instruction. The multi-locus sequence typing system (MLST; *hcpA*, *ftsZ*, *gatB*, *coxA*, and *fbpA*) [20] and partial *Wolbachia* surface protein gene (*wsp*) were amplified using the standard polymerase chain reaction (PCR) with inclusion of proper positive control and blank (ddH_2_O) in every batch of PCR reaction. Colonies were only considered to be *Wolbachia*-infected if at least one of the three assayed worker ants displayed an amplificon of the *wsp* gene with the expected size. The MLST loci were amplified following the protocols described in the PubMLST [21], whereas the *wsp* gene was amplified using PCR conditions reported in [22]. Both PCR reactions were performed using EmeraldAmp^®^ MAX PCR Master Mix (Takara, Shiga, Japan). Only one of the three randomly selected ant workers from each *Wolbachia*-positive colony was subject to Sanger sequencing and further genetic analyses (e.g., determination of mitochondrial haplotype and ddRAD sequencing, see below for more details). We assigned sequence type (ST; a unique series of alleles) for alleles of each of the MLST loci based on comparison against those deposited in the MLST database. The sequencing electropherogram of each gene was manually checked by naked eyes to identify potential infection by multiple strains of *Wolbachia*.

Host mitochondrial cytochrome *c* oxidase subunit I (*COI*) haplotype was identified from the same *A*. *gracilipes* workers subjected to Sanger sequencing for MLST loci and the *wsp* gene. Partial mitochondrial *COI* gene was amplified with primers designed based on the *A. gracilipes* mitochondrial genome [23] using software Primer3-Plus [24]. The amplification was performed using EmeraldAmp^®^ MAX PCR Master Mix (Takara, Shiga, Japan). PCR primers and conditions were listed in Table S2. The amplicons were purified and then sent to Sanger sequencing.

### 2.2. *Wolbachia* Draft Genome and ddRAD-Seq

#### 2.2.1. De Novo Assembly of *Wolbachia* Draft Genome

The MLST loci are generally conserved and may possess insufficient resolution for reconstructing recent evolutionary history of *Wolbachia* [25,26]. A *Wolbachia* draft genome was therefore assembled as a reference for uncovering more genetic variations. A female alate of *A*. *gracilipes* was collected from Taiwan (Hsinchu City, 24°46′43.43″ N, 120°56′30.24″ E) in 2014, and its high molecular weight DNA (a whole-body extraction) was purified via the standard phenol–chloroform purification method. The whole genomic DNA was sequenced by Sequencing Technology Company (Taipei, Taiwan) on the Illumina HiSeq 2500 platform with PCR-free library construction (average library insert size: 250 bp; paired-end read length: 2 × 125 bp). Illumina adapters, low-quality bases (quality score <28; base call accuracy <99.8%), and short reads (<36 bp after trimming) were trimmed using Trimmomatic v. 0.36 [27]. The whole genome de novo assembly was conducted using IDBA-UD [28] with the minimum 10 reads supported (--min_support 10; other parameters: default). The *Wolbachia* sequences were then separated from host genome via mega-BLASTn [29] against two complete supergroup A *Wolbachia* genomes [*w*Ri, host: *Drosophila simulans* Riverside (GenBank accession: GCA_000022285.1); *w*Mel, host: *Drosophila melanogaster* (GenBank accession: GCF_000008025.1)]. The contigs with e-value lower than 1e-20 were kept as our *Wolbachia* draft genome (hereinafter referred to as *w*Agra) except those with length <250 bp which were excluded from the analysis. The completeness of genome assembly was evaluated using the BUSCO v. 4.0.5 [30], which measures the proportion of highly conserved, single copy orthologs derived from 2327 proteobacterial species (proteobacteria_odb10, 219 BUSCO orthologs). The BUSCO analysis was also conducted on other supergroup A *Wolbachia* genomes (*w*Mel, *w*Ri, *w*Ha, and *w*Au) for comparison purposes. The assembly statistics were calculated using QUAST [31].

#### 2.2.2. ddRAD-Seq and *Wolbachia* SNP Filtering

The restriction site associated DNA sequencing (RAD-seq) has been demonstrated as a powerful population genomic approach for recovering high numbers of single nucleotide polymorphisms (SNPs) in various systems, especially for non-model organisms [32]. We conducted double digest RAD-seq (ddRAD-seq) on the same individuals mentioned above following the protocol described in Peterson et al. [33] with slight modifications. The restriction enzymes HpyCH4IV (New England BioLabs, Ipswich, MA, USA) and BfaI (New England BioLabs, Ipswich, MA, USA) were used for DNA digestion. In brief, 50–100 ng DNA digested with enzymes for 3 h and ligated to 5 bp inline barcodes and partial Illumina adapter at both ends with T4 DNA ligase (New England BioLabs, Ipswich, MA, USA) at 23 °C for 30 min. Ligated DNA was mixed into a DNA pool at one to one ratio. The DNA mixture was purified and concentrated using AMPure XP magnetic beads (Beckman Coulter, Pasadena, CA, USA). We then performed size selection (550–700 bp) on the condensed DNA in 2% agarose gel (Invitrogen, Carlsbad, CA, USA) and purified the selected DNA fragments using the FastGene gel/PCR extraction kit (Nippon genetics, Tokyo, Japan). The complete Illumina adapter and index were added through 12 cycles of two-step PCR enrichment (98 °C for 30 s, 12 cycles of 98 °C for 10 s and 72 °C for 30 s, with the final extension at 72 °C for 10 min, and hold at 4 °C) with Phusion high-fidelity PCR kit (New England BioLabs, Ipswich, MA, USA). The PCR library was purified using AMPure XP magnetic beads (Beckman Coulter, Pasadena, CA, USA). The final library was mixed with 10% PhiX and sequenced in the Illumina HiSeqX platform in pair-end (2 × 150) mode by Macrogen Japan (Kyoto, Japan).

Quality of generated raw reads was first examined using FastQC v. 0.11.8 [34]. To keep read quality at the same read length for SNP calling, reads containing 3′ adapters were discarded. Reads were trimmed by 1 bp on their 5′ end for both R1 and R2 reads, and by 30 bp from their 3′ end of R2 reads. Trimmed reads then were demultiplexed using 5′ anchored primer method without allowance for error (-e 0.01, --no-indels) performed in Cutadapt v. 2.8 [35]. Variant calling was conducted using a reference-based method. Reads were mapped to the *w*Agra genome using BWA v. 0.7.17 [36], and variants were called using gstacks implemented in Stacks2 v. 2.5 [37]. To minimize false positive variant call, mapped SNP loci without proper pairs were discarded (--rm-unpaired-reads). SNP loci present in more than 80% of all the samples (-R 0.8) were kept using populations program in Stacks2 v. 2.5 [37]. Employing VCFtools v. 0.1.15 [38], the SNPs with read depth less than 3 (--minDP 3) or over three times the standard deviation of SNP read depth (--maxDP 90) were identified and thus excluded from further analyses.

To verify if the SNPs we recovered were actually located in the *w*Agra genome instead of the host genome (e.g., horizontal gene transfer from *Wolbachia* to host [39]), we selected 10 polymorphic SNP loci and conducted locus-specific PCR amplification on both *Wolbachia*-infected and *Wolbachia*-free colonies. Primers were designed using Primer3-Plus [24], and the PCR conditions were accessible in the Table S2.

### 2.3. Phylogenomic Tree Reconstruction and Mitochondrial Network Analysis

The concatenated SNPs were generated by populations program in Stacks2 v. 2.5 [37]. Since the SNPs were located in various genomic regions with different mutation rates, the Jukes–Cantor model was therefore used in the analysis. The maximum likelihood phylogenetic tree was reconstructed using RAxML-NG v. 0.9.0 [40] with 1000 bootstrap replications. The mitochondrial *COI* sequences were aligned in MEGA 7 [41]. The haplotypes and network analysis were conducted in POPART [42] using the median-joining network analysis.

### 2.4. *Wolbachia* Transcriptome Analysis

The genomic regions containing polymorphic SNPs in *w*Agra were manually examined using IGV v. 2.3.71 [43]. The SNP-containing open reading frames (ORF) were annotated using NCBI ORFfinder, and potential gene function was assigned using BLASTp query against NCBI non-redundant protein sequences (nr) database. The synonymous and non-synonymous mutations were manually examined in each ORF.

To verify if the *Wolbachia* ORFs containing non-synonymous SNPs were expressed in *w*Agra, the transcriptome sequencing by RNA-Seq on *w*Agra was carried out. The RNA pool-seq was conducted on a mixture of total RNA from two adult worker ants of *A*. *gracilipes* collected in 2018. One adult worker ant was collected from Penang, Malaysia (5°21’32.4″ N, 100°18’09.0″ E), whereas the other was from Okinawa, Japan (26°40’19.2″ N, 128°00’41.0″ E). The entire worker ants were soaked in TRIzol™ RNA Extraction Reagent (Invitrogen, Carlsbad, CA, USA) and homogenized with a pestle, with the rest of the procedure following the standard TRIzol RNA extraction protocol. The RNA quality and quantity were measured with Nanodrop spectrophotometers, and then mixed at one to one ratio of the total RNA amount. The mixed sample was submitted to the Eurofins Genomics (Tokyo, Japan) where the total RNA was purified using polyA selection method, and sequencing library was constructed with the TruSeq^®^ Stranded mRNA Library Prep (insert size: 200 bp). The library was sequenced using Illumina HiSeq 4000 platform in the pair-end mode (2 × 100 bp). Read adapters and low-quality bases (base call accuracy <99.8%; q < 28) were trimmed using Trimmomatic v. 0.36 [27] and mapped onto the *w*Agra genome used HISAT2 v2.1.0 [44]. The alignment was sorted in SAMtools v. 1.9 [45] and visualized using IGV v. 2.3.71 [43]. The RNA reads in genomic regions of MLST and *wsp* gene were examined for confirming expression level of the selected *Wolbachia* genes (see Results 3.4).

### 2.5. Test for Signature of Selection

To detect the signal of selection, two *Wolbachia* genes with non-synonymous mutations (a hypothetical protein, HP_12890 and *RluA*-like gene, see Section 3.4 for more details) were amplified and sequenced for all *Wolbachia*-infected colonies. Sequences were aligned for identification of haplotype identity, and the pairwise codon-based Z-test was performed in MEGA7 for the detection of selection [41].

## 3. Results

### 3.1. Prevalance and Strain Identity of *Wolbachia*

We screened the presence of *Wolbachia* in a total of 80 *A*. *gracilipes* colonies from 12 geographical regions and revealed that the infection rates ranged from 77.78% (*n* = 7/9; Malaysia), 83.33% (*n* = 5/6; Hawaii), to 100% (other geographic regions; Table 1), resulting in a 96.25% overall infection rate (*n* = 77/80). *Wolbachia* detected in our *A*. *gracilipes* samples shared identical sequence at both MLST loci (ST52; *gatB* 22, *coxA* 2, *hcpA* 51, *ftsZ* 32, and *fbpA* 36) and the *wsp* gene (*wsp* 55, HVR1 39, HVR2 1, HVR3 44, and HVR4 40). All the sequences indicated that this *Wolbachia* strain belongs to supergroup A. We found no evidence for co-infection with more than one *Wolbachia*-variant (strain) in all infected individuals, suggesting single *Wolbachia* infection in this ant.

### 3.2. *Wolbachia* Genome and SNP Discovery

We assembled the draft genome of *Wolbachia* in *A*. *gracilipes* (*w*Agra) as the reference genome (GenBank accession: JACRYZ000000000). The draft genome assembly (96 contigs) contained 1,202,684 bp, a comparable size to other supergroup A *Wolbachia* genomes (genome size: 1.26–1.8 Mb) and within the range of other *Wolbachia* belonging to other supergroups discovered to date (supergroup B-F; 0.96–1.8 Mb). Similar to other supergroup A *Wolbachia*, GC content in the *w*Agra assembly was 35.23% (35.2–35.3%); it, however, differed from the rest of contigs with the majority of which deriving from the host ant genome (GC: 33.78%). The average genome coverage was 278.22 reads per nucleotide.

The BUSCO score of the *w*Agra genome was 85.4%, as 187 out of 219 complete and single copy proteobacteria orthologs were found in our assembly. The same BUSCO analysis was used to calculate other complete *Wolbachia* supergroup A genomes. The BUSCO scores were 84.5% in *w*Mel and *w*Ri, 84% in *w*Ha, and 84.9% in *w*Au, suggesting that our assembly, despite being fragmented, has recovered most of the proteobacteria orthologs. Thus, the assembly could serve as the reference genome with robustness to reveal the population genomic variations of this bacterium (Table 2).

Our SNP calling and filtering yielded 129 SNP loci from 68 *w*Agra-infected individuals (a total of 12 individuals were excluded from the analysis, and these individuals included 3 *Wolbachia*-free individuals and 9 individuals that failed to pass our SNP filtering). We found 53 SNP loci that possess polymorphisms. To verify if the discovered SNPs were located in the *Wolbachia* genome, we randomly selected 10 SNP loci and PCR-amplified on both *Wolbachia*-infected and *Wolbachia*-free colonies. All 10 polymorphic SNP loci were successfully amplified only from *Wolbachia*-infected samples but not *Wolbachia*-free ones, suggesting that these SNPs originated from the *w*Agra genome.

### 3.3. Phylogenomic Tree and Mitochondrial Network

**Figure 2:**
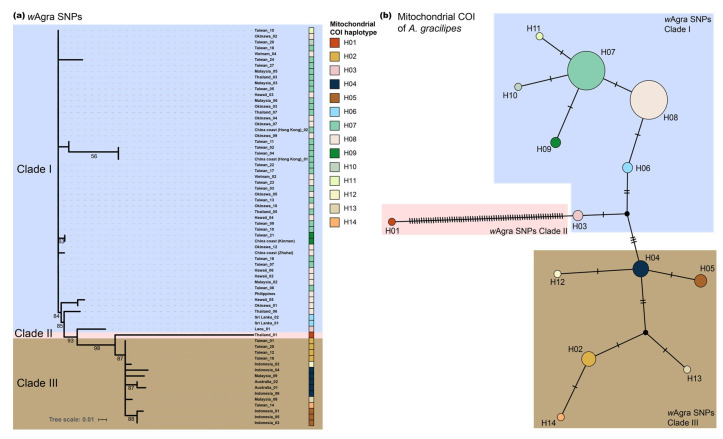
(**a**) *Wolbachia* (*w*Agra) SNPs phylogenetic tree based on Maximum Likelihood method. The mitochondrial *COI* haplotype of each sample is indicated in color boxes following individual name. Numbers at node indicate bootstrap support values (1000 replicates). Tree branches with <50% bootstrap supporting value were removed. (**b**) The median-joining network analysis of *A*. *gracilipes* mitochondrial *COI* haplotypes. The color shading depicts the *w*Agra SNPs clade displayed in (**a**). Circle area is proportional to the number of individuals carrying a given haplotype.

We compared the phylogenetic trees based on 129 *Wolbachia*-origin SNPs and the network analysis based on partial mitochondrial *COI* sequence of the host (854 bp; GenBank accessions: MT899004-MT899082) and found a high degree of concordance (Figure 2). *w*Agra from Thailand (Thailand_01) was among the most genetically divergent (Figure 2a), coinciding with the fact that mitochondrial *COI* haplotype from the same individual was characterized with the most nucleotide polymorphisms among all the samples (Figure 2b). Individuals harboring mitochondrial haplotypes H06-H11 were grouped together in *Wolbachia* SNPs Clade I (Figure 2; blue square), whereas the remaining samples including those from Indonesia, Australia, Malaysia (Borneo), and part of southern Taiwan were clustered in *Wolbachia* SNPs Clade III (Figure 2; brown square). To exclude the possibility of the observed similar topology resulting from amplification of the *Wolbachia COI* instead of the host mitochondrial *COI*, we used BLASTn (task: blastn-short) to compare *COI* primers against the *w*Agra genome, and the result indicated no *Wolbachia* amplification was available. We also compared mitochondrial *COI* sequences against NCBI database with BLASTn and verified the *COI* sequences we amplified indeed derived from *A*. *gracilipes*.

### 3.4. Gene Expression and Non-Synonymous ORFs

Alignment of the *Wolbachia* SNPs revealed that eight distinct SNP loci contributed to the separation of *Wolbachia* SNPs Clade I and Clade III. To further dissect the SNP polymorphisms associated with selection and/or demography, we analyzed the synonymous and non-synonymous mutations of these SNP loci. Seven of the eight SNP loci were located in ORFs, and three out of these seven were non-synonymous mutations, with two of which each residing in one of two *Wolbachia* hypothetical proteins and one in *RluA*-like gene. The *RluA* is a pseudouridine synthase gene involved in RNA binding and has been known to participate in the cellular information processing [46].

To verify if the genes with non-synonymous mutation express in *w*Agra, we examined the transcriptome from a pooled RNA sample prepared from two yellow crazy ant workers. In total, 32,771,008 paired RNA-seq reads passed the quality control. Among these, 11,975 (0.04%) of reads mapped onto the *w*Agra draft genome assembly. First, we found the MLST genes had fewer reads (*coxA*: 0, *hcpA*: 2, *gatB*: 4, *fbpA*: 8, and *ftsZ*: 26 reads per gene) than the *wsp* gene (73 reads). We then inspected the non-synonymous mutation containing ORFs and found that the hypothetical protein (scaffold_12890: 3880–5208 bp, hereinafter referred to as HP_12890; GenBank accession: MT896046) possessed 70 RNA reads, which is comparable to that of the *wsp* gene. Another hypothetical protein (scaffold_2912: 3397–3696 bp) had only 9 reads, whereas no evidence of expression of the *RluA*-like gene (GenBank accession: MT896124) was detected.

### 3.5. Adaptative Selections

**Figure 3:**
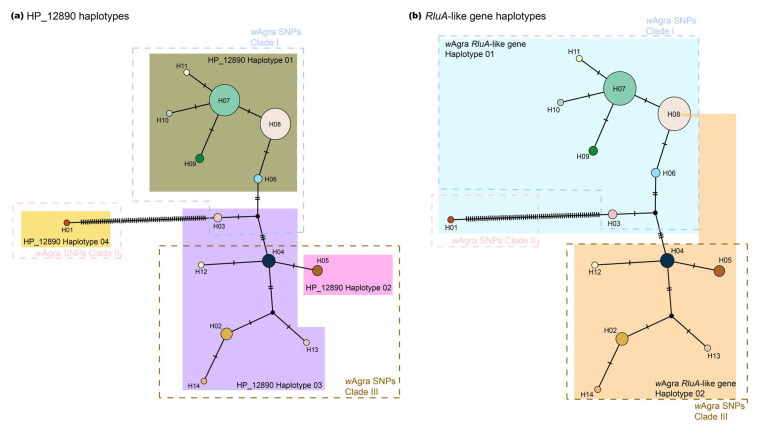
Mitochondrial *COI* haplotypes of *A*. *gracilipes* (circles in color) and their corresponding *Wolbachia* (**a**) HP_12890, and (**b**) *RluA*-like gene haplotypes. Dashed lines indicate the *w*Agra SNPs clade shown in Figure 2a. Circle area is proportional to the number of individuals carrying a given haplotype.

We sequenced partial fragment of HP_12890 and *RluA*-like gene from all *Wolbachia*-infected colonies. HP_12890 possessed four haplotypes (Figure 3a; GenBank accessions: MT895969- MT896045), whereas *RluA*-like gene had two haplotypes (Figure 3b; GenBank accessions: MT896047- MT896123). Both genes harbored the SNP(s) that were detected by ddRAD-seq. Moreover, haplotype distribution of HP_12890 was generally concordant to the *Wolbachia* SNPs clades. For example, the haplotype I of HP_12890 was found in most of individuals belonging to *Wolbachia* SNPs Clade I (Figure 3, highlighted in olive green square, mitochondrial H06-H11); while haplotype II of HP_12890 was restricted to individuals harboring mitochondrial *COI* haplotype V (H05). The haplotype III of HP_12890 is associated with individuals bearing mitochondrial *COI* haplotype H02-H04 and H12-H14, and the haplotype IV of HP_12890 was only harbored in Thailand_01 with a unique mitochondrial *COI* haplotype (H01; Figure 3). Among the four HP_12890 haplotypes, we found three polymorphic sites with all conveyed by non-synonymous mutations.

A similar pattern was found in the haplotype distribution of *RluA*-like gene, with individuals from *Wolbachia* SNPs Clade I generally harboring one haplotype (*RluA*_H01) and those from Clade III harboring the other haplotype (*RluA*_H02). One exception, however, involved an individual in China (Yunnan) with the *RluA*-like haplotype that was found in the individual belonging to *Wolbachia* SNPs Clade III but harboring mitochondrial *COI* haplotype H08 (*Wolbachia* SNPs Clade I). The only difference between the two *RluA*-like gene haplotypes was a non-synonymous mutation.

Results of the Z-test indicated the presence of signature of positive selection (dN > dS; *p* = 0.043) on the HP_12890 haplotype IV in Thailand (Thailand_01) compared to the most common HP_12890 haplotype (haplotype I, mitochondrial *COI* haplotype H06-H11). Signal of positive selection was not detected in the *RluA*-like gene ORF (scaffold_2786:4747-5286 bp; *p* = 0.16). We also found no evidence for purifying selection among all haplotype pairs for both genes (dN < dS; *p* = 1).

In addition, we compared HP_12890 against NCBI nucleotide collection (nr/nt) database with mega-BLASTn, and showed that orthologs of HP_12890 were present in *Wolbachia* of blowflies (*Chrysomya megacephala*; nucleotide identity: 93% (704/758); GenBank accession: CP021120.1) and the Asian citrus psyllid (*Diaphorina citri*; nucleotide identity: 91% (700/770); GenBank accession: CP051608.1). The results of interspecific Z-test indicated evidence of purifying selection for HP_12890 (dN < dS; *p* < 0.001), while there was no evidence of positive selection between species (dN > dS; *p* = 1). A similar pattern was found for *RluA*-like gene: we compared *Wolbachia* in hosts, including the horn fly (*Haematobia irritans*; *w*Irr; nucleotide identity: 96% (541/566); GenBank accession: CP037426.1) and fruit fly (*Drosophila melanogaster*; *w*Mel; nucleotide identity: 92% (523/567); GenBank accession: CP042445.1), and the interspecific Z-test was significant for purifying selection (dN < dS; *p* < 0.001) but not positive selection (dN > dS; *p* = 1).

## 4. Discussion

### 4.1. Origin of *Wolbachia* in Yellow Crazy Ant

Our study extended the findings of Tseng et al. [18] and Sebastien et al. [19] showing that all yellow crazy ants across broad geographical regions harbor *Wolbachia* with identical sequences of both housekeeping (e.g., MLST) and fast-evolving genes (e.g., *wsp*), suggesting that evolutionary history of *w*Agra should be recent and that *w*Agra may have derived from a single ancestral population. The MLST strain (ST52) in the yellow crazy ant (Formicinae) was also reported in an ant *Lophomyrmex* sp. (Myrmicinae) in Thailand [47]. Considering the fact that divergence time of *Lophomyrmex* sp. and *A. gracilipes* is approximately 111 MYA (CI: 99-126 MYA) [48], identical MLST sequences may imply the occurrence of horizontal transfer between the two ant species. Since phylogenies of *Wolbachia* and the ant’s mitochondrial *COI* mirror to each other, it is likely that there may have been no additional horizontal transfer of *Wolbachia* involving other ant species bearing different MLST strains along the evolutionary time. Tseng et al. [18] showed that horizontal transfer of *Wolbachia* is prevailing between ant and ant guests with an intimate ecological association (e.g., longhorn crazy ant, *Paratrechina longicornis* and its host-specific ant cricket, *Myrmecophilous americanus*), except *A. gracilipes* and its closely-associated, host-specific ant cricket, *M*. *albicinctus*. Combined with near fixation of *Wolbachia* in all sampled *A. gracilipes* populations in this study, vertical transmission, rather than horizontal, appears to be the predominant route for the spread of *Wolbachia* within this ant species.

### 4.2. High Infection Rate with a Single *Wolbachia* Strain

One major finding of the present study is that *Wolbachia* is highly prevalent across sampled colonies of *A*. *gracilipes* in virtually all geographic regions. In some cases, *Wolbachia* infection rates in ants were found to be 100%, but most of these studies either were conducted at a rather local scale (e.g., single collection site) [49] or that the infection rates fluctuate temporally and spatially [22,47,50,51,52,53,54,55,56]. A similar *Wolbachia* prevalence pattern has been reported in the parasitic wasp [57], bedbugs [5], and filarial nematodes [3,7]. These hosts apparently rely on *Wolbachia* for essential functions such as nutritional provision, metabolism facilitation, or oocyte maturation and thus engage in an exclusive mutualistic relationship with *Wolbachia*. Hence, the observed high prevalence of *Wolbachia* in *A. gracilipes* may imply an intimate association between *Wolbachia* and *A. gracilipes*, although more empirical tests are needed.

The selection pressures exerted by natural enemies often lead to an increase of particular inherited symbionts in insect populations [58,59]. For example, high infection of *Spiroplasma* is observed in *Drosophila* populations with the presence of parasitic nematodes [60]. While natural enemies of *A. gracilipes* and their pathogenicity remain poorly described (as compared to other major pest insects), several potential pathogens (i.e., Anoplolepis gracilipes virus 1, Anoplolepis gracilipes virus 2, and TR44839 virus) have been reported in Cooling et al. [61] and Lee et al. [62]. Evidence is emerging to suggest some of which can be pathogenic and associated with fitness cost of the host [63]. TR44839 virus was found to be highly prevalent in *A. gracilipes* populations in Okinawa (Japan), Taiwan, and Penang (Malaysia) [64], it, however, appears to persist in low viral titers (Hsu et al., unpublished data). The two dicistroviruses (Anoplolepis gracilipes virus 1 and Anoplolepis gracilipes virus 2) were also reported in the ant collected from Taiwan and Malaysia [62]. High prevalence of *Wolbachia* may potentially be maintained by benefits of endosymbiotic-driven defense that provides *A. gracilipes* protection against these viral pathogens, as this is the case frequently observed in flies [65] and mosquitos [66].

Another likely explanation for the high prevalence of *Wolbachia* in *A*. *gracilipes* is “Jekyll and Hyde” infection of *Wolbachia* [2,67]. *Wolbachia* may act as beneficial symbiont and reproductive parasite simultaneously, which is termed “Jekyll and Hyde” infection [2,67], and this may largely facilitate the endosymbiont spread in the host population [67,68]. For example, rapid spread and high infection frequency of *Wolbachia* in California *Drosophila* populations were shown to be associated with the dynamic interaction between parasitic and mutualistic life modes of *Wolbachia* [69]. The reproductive manipulation of *Wolbachia* in ants seems uncommon (but see [13], with the effects, if any, being highly species-dependent). For example, *Wolbachia* infection possesses negligible influence on sex ratio in Formica ants [70,71] and yet drives the Pharaoh ant *Monomorium pharaonis* toward female-biased [12]. Interestingly, the same *Wolbachia* strain was also found to promote colony growth and early colony reproduction of the Pharaoh ant [72], providing arguably the first case of “Jekyll and Hyde” infection of *Wolbachia* in ant. Whether *w*Agra manipulates reproduction and/or enhance productivity of *A*. *gracilipes* remains an open question, and empirical evidence is now being generated.

Host life-history style may represent another potential reason for the extraordinarily high prevalence of *Wolbachia* in *A*. *gracilipes*. For example, *Wolbachia* infection rate in beetles is associated with reproduction mode (e.g., parthenogenesis), mobility, geographical distribution, body size, and/or population connectivity (e.g., fragmented or isolated) [73]. In ants, species with polygynous colony structure (multiple reproductive queens) and budding as major colony reproduction mode generally harbor a higher *Wolbachia* infection rate, and these traits may have facilitated the persistence of *Wolbachia* in the host [74]. We, however, failed to find evidence for such support, as other ants with a similar life-history style were never reported to have an even comparable level of *Wolbachia* prevalence observed in *A*. *gracilipes*.

### 4.3. Evidence for *Wolbachia*–Host Coevolution

While it remains uncertain that *Wolbachia* are in parasitic or mutualist association with *A*. *gracilipes*, *Wolbachia*–host coevolution should be expected, given the extremely high *Wolbachia* infection in the ant and the high level of concordance between *Wolbachia* SNPs phylogenetic tree and geographical distribution of mitochondrial *COI* haplotypes of *A*. *gracilipes*.

Gene flow between different supercolonies of *A. gracilipes* is limited because a high level of aggression is expected between workers (and possibly between worker and gyne) originating from different supercolonies, and this pattern applies even within a fine geographical scale [16,17,64]. Combined with the fact that independent foundation is rare in *A*. *gracilipes* [15], spread of *Wolbachia* at a local scale is either discouraged or highly dependent on supercolony structure. Nevertheless, human-mediated jump dispersal has been the primary mode of spread for many invasive ants [75] including *A*. *gracilipes*. Indeed, historical records and our preliminary analyses (Lee et al., unpublished data) showed that *A*. *gracilipes* has been transported to multiple Indo-Pacific islands through anthropogenic activities [14,76]. Such a strong affinity with human-associated transportation is predicted to create a route for its associated symbionts, including *Wolbachia*, to spread across different biogeographical regions. Co-divergence of *Wolbachia* and mitochondrial *COI* of *A*. *gracilipes* provides firm support to this prediction and suggests that this bacterium may have frequently spread with the host ant as a hitchhiker. An alternative possibility is that invasion success of this ant is at least partially associated with *Wolbachia* infection (e.g., *Wolbachia* as a potential nutritional mutualist in introduced populations of the ghost ant, *Tapinoma melanocephalum* [77]). This possibility is of particular interest in the context of invasion biology as our finding represents one of very few cases, especially in invasive ants, in which *Wolbachia* persists at high prevalence across the entire invasive range. Several factors—including drift, altered selection pressures, imperfect maternal transmission, or natural curing events—are attributable to the loss (or low prevalence) of *Wolbachia* infection during colonization of invasive ants [56,78,79], yet our results provide a new research avenue to investigate how interplay between *Wolbachia* and host shapes the invasion success of ant.

We note that one particular sample from Thailand (Thailand_01) possesses a deep genetic divergence in both *Wolbachia* and host mitochondria DNA from other samples of *A*. *gracilipes* (Figure 2), suggesting there is under-discovered yet great deal of genetic diversity in or somewhere near the western part of Thailand and that the populations in this region might be the origin of this invasive ant. Such a finding is parallel to the Southeast Asian origin of *A*. *gracilipes* proposed by multiple studies [14,15] despite more comprehensive sampling and additional nuclear data needed. Furthermore, it has been demonstrated that endosymbionts could assist in disclosing the hidden and recent population demography of its host [80,81], combined with the fact that *Lophomyrmex* ants in Thailand and *A*. *gracilipes* share an identical MLST [47], characterization of genetic variation of *Wolbachia* in both ant species in the particular region would accelerate reconstructing the origin of *Wolbachia*, as well as their hosts through understanding the coevolutionary history.

### 4.4. Rapid Evolution of a Hypothetical *Wolbachia* Gene

The observed signature of co-divergence between *w*Agra and the host ant *A*. *gracilipes* raises a question of whether it is driven by the bacterium or simply reflects the demographic history of host population. We confirmed the presence of non-synonymous mutations in the two *Wolbachia* genes, and one of which, namely *RluA*-like gene, is crucial for bacterial cellular functions [46]. Signal of purifying selection revealed by the interspecific Z-test supports the essential role of this gene in *Wolbachia*. While we observed nucleotide polymorphisms in *w*Agra across our sampled populations of *A*. *gracilipes*, no evidence for expression nor signature of positive selection suggests that these nucleotide polymorphisms are most likely associated with demographic history of the host such as bottleneck during population expansion or genetic drift, although we cannot rule out the possibility that divergence time was not sufficient to accumulate synonymous mutations or that our RNA sequencing depth is insufficient to detect the evidence for expression.

We fully understand that the read number here serves an indicator of “relative” abundance of gene expression because our RNA sequencing read depth likely is below the level where gene expression pattern can be precisely characterized. However, our transcriptome analysis verifies the expression of HP_12890 in *w*Agra and shows that HP_12890 possesses comparable RNA reads to the *wsp* gene that plays a key role in mediating *Wolbachia*–host interactions. One potential explanation for such high expression level is that HP_12890 may interact intimately with its host *A*. *gracilipes*. Alternatively, it is likely that HP_12890 acts as a selfish element (e.g., mobile elements), and the observed polymorphism may be the result of selfish gene replication, given that the selfish element usually can be highly expressed and spreads rapidly in the host genome [82]. We, however, did not detect the presence of multiple copies, a typical characteristic of selfish element, nor sequence polymorphisms within individuals in our analysis. Interestingly, the Z-test indicates signature of purifying selection in interspecific comparison but positive selection among intraspecific haplotypes for HP_12890. This suggests that this gene may be functionally important and that the observed non-synonymous mutations may be associated with host demography and/or rapid evolution of *Wolbachia*.

## 5. Conclusions

This study reports a single *Wolbachia* strain that is nearly fixed in a globally distributed invasive ant species and adds to the existing literature showing *Wolbachia*–ant coevolution. More importantly, our data may represent a rare case involving *Wolbachia* as a potential mutualist in ants and raises an invasion biology-centered question: how this bacterium is maintained at high prevalence during the colonization of this ant. Genetic and functional characterization of *Wolbachia* and its phenotypic effect on *A*. *gracilipes* would offer additional insights into the dynamics of the *Wolbachia*–ant coevolution.

## Figures and Tables

**Table 1:** *Wolbachia* prevalence in *Anoplolepis gracilipes* across all sampled regions.

Geographical Region	No. of Infected Colony	No. of Uninfected Colony	Infection Rate
Okinawa	12	0	100.00%
Hawaii	5	1	83.33%
Taiwan	26	0	100.00%
Southeast coastal China (Hong Kong and Zhuhai) and Kinmen Island, Taiwan	4	0	100.00%
Laos and Southwest China (Yunnan)	2	0	100.00%
Vietnam	3	0	100.00%
Thailand	7	0	100.00%
Sri Lanka	2	0	100.00%
Philippines	1	0	100.00%
Malaysia	7	2	77.78%
Indonesia	6	0	100.00%
Australia	2	0	100.00%
Total	77	3	96.25%

**Table 2:** Statistics of *Wolbachia* genome assembly.

Strain	Host Species	Super- Group	No. of Contig	Genome Size (Mb)	GC%	BUSCO Score (%)	RefSeq Assembly Accession
*w*Au	*Drosophila simulans*	A	1	1.26	35.2	84.9	GCF_000953315.1
*w*Ha	*Drosophila simulans*	A	1	1.29	35.3	84	GCF_000376605.1
*w*Mel	*Drosophila melanogaster*	A	1	1.27	35.2	84.5	GCF_000008025.1
*w*Ri	*Drosophila simulans*	A	1	1.45	35.2	84.5	GCF_000022285.1
*w*Agra	*Anoplolepis gracilipes*	A	96	1.2	35.2	85.4	JACRYZ000000000 (This study)

BUSCO scores for all genomes were calculated with identical parameters using proteobacteria_obd10.

## Supplementary Materials

The following are available online at https://www.mdpi.com/2076-2607/8/10/1569/s1, Table S1: Sample information of *Anoplolepis gracilipes* and detailed profile of associated *Wolbachia*, Table S2: Primers and PCR conditions used in this study.
