# Supplementary material for: Ongoing Coevolution of Wolbachia and a Widespread Invasive Ant, Anoplolepis gracilipes
Source: Microorganisms. 2020 Oct 12;8(10):1569. doi: 10.3390/microorganisms8101569 (PMC7601630; doi:10.3390/microorganisms8101569)
Supplement: Supplementary file 1 [file microorganisms-08-01569-s001.pdf]

Table S1. Sample information of *Anoplolepis gracilipes* and detailed profile of associated *Wolbachia*

Sample ID	Region	GPS (Latitude)	GPS (Longtitude)	*A . gracilipes* mitochondrial COI haplotype	*A . gracilipes* mitochondrial COI GenBank accession numbe	*Wolbachia* MLST	*w* Agra SNPs clade	*Wolbachia hypothetical protein* (HP_12890) haplotype	*Wolbachia hypothetical protein* (HP_12890) GenBank accession numbe	*Wolbachia RluA* -like gene haplotype	*Wolbachia RluA-like* gene GenBank accession numbe	*A. gracilipes* collection date
Australia_01	Australia	-17.04561699	145.729007	H04	MT899010	ST52	Clade 3	H03	MT896037	H02	MT896116	16-Jul-2014
Australia_02	Australia	-17.05242871	145.7296556	H04	MT899011	ST52	Clade 3	H03	MT896038	H02	MT896117	16-Jul-2014
China (Yunnan)	China (Yunnan)	21.920641	101.277187	H08	MT899048	ST52	Unassigned	H01	MT895998	H02	MT896108	21-Jan-2015
SE cosatal China (Hong Kong) 01	China Coast	22.283441	114.136937	H07	MT899020	ST52	Clade 1	H01	MT895971	H01	MT896049	8-Mar-2018
SE cosatal China (Hong Kong) 02	China Coast	22.282944	114.133855	H07	MT899021	ST52	Clade 1	H01	MT895972	H01	MT896050	8-Mar-2018
Kinmen Island, Taiwan	China Coast	24.434463	118.316986	H09	MT899076	ST52	Clade 1	H01	MT896024	H01	MT896101	4-Jul-2015
SE cosatal China (Zhuhai)	China Coast	22.2493662	113.5491724	H08	MT899049	ST52	Clade 1	H01	MT895999	H01	MT896076	1-Jan-2016
Hawaii_01	Hawaii	21.271137	-157.69707	H08	MT899050	Uninfected	Uninfected	-	-	-	-	23-Aug-2013
Hawaii_02	Hawaii	19.656832	-155.009814	H08	MT899051	ST52	Clade 1	H01	MT896000	H01	MT896077	25-Aug-2013
Hawaii_03	Hawaii	19.428898	-155.258168	H08	MT899052	ST52	Clade 1	H01	MT896001	H01	MT896078	27-Aug-2013
Hawaii_04	Hawaii	19.686359	-155.070893	H08	MT899053	ST52	Clade 1	H01	MT896002	H01	MT896079	28-Aug-2013
Hawaii_05	Hawaii	19.80746	-155.09387	H08	MT899054	ST52	Clade 1	H01	MT896003	H01	MT896080	29-Aug-2013
Hawaii_06	Hawaii	21.2071106	-157.9073066	H08	MT899055	ST52	Clade 1	H01	MT896004	H01	MT896081	29-Aug-2013
Indonesia_01	Indonesia	-6.821408	107.556061	H05	MT899015	ST52	Clade 3	H02	MT896028	H02	MT896109	Aug-2013
Indonesia_02	Indonesia	-8.39217	115.29561	H12	MT899080	ST52	Clade 3	H03	MT896042	H02	MT896121	3-Aug-2014
Indonesia_03	Indonesia	-6.588447	106.804277	H05	MT899016	ST52	Clade 3	H02	MT896029	H02	MT896110	3-Nov-2017
Indonesia_04	Indonesia	-7.299701	112.740204	H04	MT899012	ST52	Clade 3	H03	MT896039	H02	MT896118	Dec-2014
Indonesia_05	Indonesia	-6.595687	106.796705	H05	MT899017	ST52	Clade 3	H02	MT896030	H02	MT896111	17-Nov-2016
Indonesia_06	Indonesia	-6.500481	106.844315	H04	MT899013	ST52	Clade 3	H03	MT896040	H02	MT896119	Apr-2018
Laos_01	Laos	21.683	102.1	H03	MT899008	ST52	Clade 1	H03	MT896035	H01	MT896105	15-Aug-2012
Malaysia_01	Malaysia	5.64641	100.485607	H07	MT899022	Uninfected	Uninfected	-	-	-	-	20-Oct-2013
Malaysia_02	Malaysia	2.27197	102.297987	H08	MT899056	ST52	Clade 1	H01	MT896005	H01	MT896082	27-Jan-2014
Malaysia_03	Malaysia	1.841309	102.955261	H07	MT899023	ST52	Clade 1	H01	MT895973	H01	MT896051	9-Feb-2014
Malaysia_04	Malaysia	5.365015	100.394476	H08	MT899057	Uninfected	Uninfected	-	-	-	-	9-Feb-2014
Malaysia_05	Malaysia	5.356826	100.304936	H07	MT899024	ST52	Clade 1	H01	MT895974	H01	MT896052	21-Mar-2015
Malaysia_06	Malaysia	5.387794655	100.2797447	H07	MT899025	ST52	Clade 1	H01	MT895975	H01	MT896053	10-Sep-2015
Malaysia_07	Malaysia	5.152209	103.104877	H08	MT899058	ST52	Unassigned	H01	MT896006	H01	MT896083	1-Mar-2015
Malaysia_08	Malaysia	1.829	103.834	H13	MT899081	ST52	Clade 3	H03	MT896043	H02	MT896122	2-Aug-2014
Malaysia_09	Malaysia	3.42158	115.152602	H04	MT899014	ST52	Clade 3	H03	MT896041	H02	MT896120	20-Jan-2016
Okinawa_01	Okinawa	26.17226056	127.8265289	H08	MT899059	ST52	Clade 1	H01	MT896007	H01	MT896084	7-Jan-2014
Okinawa_02	Okinawa	26.22716639	127.7164883	H08	MT899060	ST52	Clade 1	H01	MT896008	H01	MT896085	7-Jan-2014
Okinawa_03 (Ishigaki)	Okinawa	24.41169096	124.2074617	H07	MT899026	ST52	Clade 1	H01	MT895976	H01	MT896054	8-Jan-2014
Okinawa_04	Okinawa	26.732522	128.169354	H08	MT899061	ST52	Clade 1	H01	MT896009	H01	MT896086	13-Jan-2014
Okinawa_05	Okinawa	26.729676	128.167866	H08	MT899062	ST52	Clade 1	H01	MT896010	H01	MT896087	13-Jan-2014
Okinawa_06	Okinawa	26.468733	127.82925	H08	MT899063	ST52	Unassigned	H01	MT896011	H01	MT896088	1-Apr-2014
Okinawa_07	Okinawa	26.14153409	127.7490789	H08	MT899064	ST52	Clade 1	H01	MT896012	H01	MT896089	5-Apr-2014
Okinawa_08	Okinawa	26.58023754	127.9853719	H08	MT899065	ST52	Unassigned	H01	MT896013	H01	MT896090	6-Apr-2014
Okinawa_09	Okinawa	26.63114293	128.1539032	H08	MT899066	ST52	Clade 1	H01	MT896014	H01	MT896091	6-Apr-2014
Okinawa_10	Okinawa	26.69422598	127.8791086	H08	MT899067	ST52	Clade 1	H01	MT896015	H01	MT896092	6-Apr-2014
Okinawa_11	Okinawa	26.69137395	127.879264	H08	MT899068	ST52	Unassigned	H01	MT896016	H01	MT896093	6-Apr-2014
Okinawa_12	Okinawa	26.68008721	127.8868282	H08	MT899069	ST52	Clade 1	H01	MT896017	H01	MT896094	6-Apr-2014
Philippines	Philippines	12.70301	124.033229	H08	MT899070	ST52	Clade 1	H01	MT896018	H01	MT896095	3-Spe-2015
Sri Lanka_01	Sri Lanka	6.9729083	79.9136483	H06	MT899018	ST52	Clade 1	H01	MT895969	H01	MT896047	26-Oct-2015
Sri Lanka_02	Sri Lanka	6.971673	79.913187	H06	MT899019	ST52	Clade 1	H01	MT895970	H01	MT896048	26-Oct-2015
Taiwan_01	Taiwan	22.458303	120.480639	H02	MT899004	ST52	Clade 3	H03	MT896031	H02	MT896112	27-Jan-2013
Taiwan_02	Taiwan	24.935841	121.188029	H07	MT899027	ST52	Clade 1	H01	MT895977	H01	MT896055	6-Feb-2013
Taiwan_03	Taiwan	23.602037	121.52006	H07	MT899028	ST52	Clade 1	H01	MT895978	H01	MT896056	27-Jan-2013
Taiwan_04	Taiwan	24.530652	120.853144	H07	MT899029	ST52	Clade 1	H01	MT895979	H01	MT896057	16-Jul-2014
Taiwan_05	Taiwan	22.014391	121.572348	H07	MT899030	ST52	Clade 1	H01	MT895980	H01	MT896058	Oct-2015
Taiwan_06	Taiwan	22.671203	121.467793	H07	MT899031	ST52	Unassigned	H01	MT895981	H01	MT896059	Oct-2015
Taiwan_07	Taiwan	24.907286	121.850072	H07	MT899032	ST52	Clade 1	H01	MT895982	H01	MT896060	16-Dec-2012
Taiwan_08	Taiwan	22.901387	121.15189	H07	MT899033	ST52	Clade 1	H01	MT895983	H01	MT896061	3-Apr-2016
Taiwan_09	Taiwan	23.485651	120.468207	H07	MT899034	ST52	Clade 1	H01	MT895984	H01	MT896062	9-Oct-2012
Taiwan_10	Taiwan	22.507511	120.587913	H07	MT899035	ST52	Clade 1	H01	MT895985	H01	MT896063	11-Feb-2013

Taiwan_11	Taiwan	24.13644895	120.6851126	H07	MT899036	ST52	Clade 1	H01	MT895986	H01	MT896064	7-Feb-2013
Taiwan_12	Taiwan	22.894924	120.622337	H02	MT899005	ST52	Clade 3	H03	MT896032	H02	MT896113	13-Feb-2013
Taiwan_13	Taiwan	25.12038	121.859601	H07	MT899037	ST52	Clade 1	H01	MT895987	H01	MT896065	28-May-2013
Taiwan_14	Taiwan	22.91338205	120.690431	H14	MT899082	ST52	Clade 3	H03	MT896044	H02	MT896123	29-Jun-2013
Taiwan_15	Taiwan	22.88697022	120.6672609	H11	MT899079	ST52	Clade 1	H01	MT896027	H01	MT896104	29-Jun-2013
Taiwan_16	Taiwan	24.226566	120.578353	H07	MT899038	ST52	Clade 1	H01	MT895988	H01	MT896066	24-Jul-2013
Taiwan_17	Taiwan	22.531149	120.963353	H07	MT899039	ST52	Clade 1	H01	MT895989	H01	MT896067	4-Aug-2013
Taiwan_18	Taiwan	25.08727	121.622726	H07	MT899040	ST52	Clade 1	H01	MT895990	H01	MT896068	16-Oct-2013
Taiwan_19	Taiwan	22.969407	120.687792	H02	MT899006	ST52	Clade 3	H03	MT896033	H02	MT896114	12-Apr-2014
Taiwan_20	Taiwan	24.572278	121.496351	H10	MT899078	ST52	Clade 1	H01	MT896026	H01	MT896103	25-Apr-2014
Taiwan_21	Taiwan	22.998438	120.218698	H09	MT899077	ST52	Clade 1	H01	MT896025	H01	MT896102	2-Aug-2014
Taiwan_22	Taiwan	24.77873	120.941733	H07	MT899041	ST52	Clade 1	H01	MT895991	H01	MT896069	12-Aug-2014
Taiwan_23	Taiwan	23.942608	121.543501	H08	MT899071	ST52	Clade 1	H01	MT896019	H01	MT896096	16-Nov-2014
Taiwan_24	Taiwan	24.179118	121.507917	H07	MT899042	ST52	Clade 1	H01	MT895992	H01	MT896070	17-Nov-2014
Taiwan_25	Taiwan	22.089839	120.716359	H02	MT899007	ST52	Clade 3	H03	MT896034	H02	MT896115	27-Sep-2014
Taiwan_27	Taiwan	22.533892	120.934388	H07	MT899043	ST52	Clade 1	H01	MT895993	H01	MT896071	27-Aug-2014
Thailand_01	Thailand	14.352859	100.532432	H01	-	ST52	Clade 2	H04	MT896045	H01	MT896107	2-Aug-2015
Thailand_02	Thailand	13.68233015	100.6597622	H07	MT899044	ST52	Unassigned	H01	MT895994	H01	MT896072	3-Aug-2015
Thailand_03	Thailand	14.58978659	101.0233643	H07	MT899045	ST52	Clade 1	H01	MT895995	H01	MT896073	4-Aug-2015
Thailand_04	Thailand	14.83454386	101.5498545	H03	MT899009	ST52	Unassigned	H03	MT896036	H01	MT896106	4-Aug-2015
Thailand_05	Thailand	14.51448021	101.9590169	H07	MT899046	ST52	Clade 1	H01	MT895996	H01	MT896074	4-Aug-2015
Thailand_06	Thailand	14.43443974	101.8743358	H08	MT899072	ST52	Clade 1	H01	MT896020	H01	MT896097	4-Aug-2015
Thailand_07	Thailand	14.371849	101.863797	H07	MT899047	ST52	Clade 1	H01	MT895997	H01	MT896075	4-Aug-2015
Vietnam_01	Vietnam	11.1437618	106.4594941	H08	MT899073	ST52	Unassigned	H01	MT896021	H01	MT896098	8-Feb-2015
Vietnam_02	Vietnam	10.774628	106.671395	H08	MT899074	ST52	Clade 1	H01	MT896022	H01	MT896099	24-Oct-2019
Vietnam_04	Vietnam	10.774628	106.671395	H08	MT899075	ST52	Clade 1	H01	MT896023	H01	MT896100	24-Oct-2019

Table S2. Primers and PCR conditions used in this study

Target species/gene	Primer name	Primer sequence (5' to 3' end)	PCR condition	Reference
Yellow crazy ant (*Anoplolepis gracilipes*)				
Anoplolepis gracilipes mitochondrial COI	YCAmt58_F<br>YCAmt1581_R	5′-TTTGCAGTTTGAGCCGGAAT-3′<br>5′-TCTATTGCACTAATCTGCCA-3′	98°C for 3 min.<br>35 cycles: 98°C for 10 sec., 55°C for 30 sec. and 72°C for 100 sec.<br>final extension at 72°C for 5 min.<br>hold at 12°C	This study
Wolbachia				
gatB	gatB_F1<br>gatB_R1	5'-GAKTTAAAYCGYGCAGGBGTT-3'<br>5'-TGGYAAYTCRGGYAAAGATGA-3'	94°C for 2 min.<br>37 cycles: 94°C for 30 sec., 54°C for 45 sec. and 72°C for 90 sec.<br>final extension at 72°C for 10 min.<br>hold at 12°C	https://pubmlst.org/wolbachia/info/protocols.shtml
coxA	coxA_F1<br>coxA_R1	5'-TTGGRGCRATYAACTTTATAG-3'<br>5'-CTAAAGACTTTKACRCCAGT-3'	94°C for 2 min.<br>37 cycles: 94°C for 30 sec., 54°C for 45 sec. and 72°C for 90 sec.<br>final extension at 72°C for 10 min.<br>hold at 12°C	
hcpA	hcpA_F1<br>hcpA_R1	5'-GAAATARCAGTTGCTGCAAA-3'<br>5'-GAAAGTYRAGCAAGYTCTG-3'	94°C for 2 min.<br>37 cycles: 94°C for 30 sec., 54°C for 45 sec. and 72°C for 90 sec.<br>final extension at 72°C for 10 min.<br>hold at 12°C	
ftsZ	ftsZ_F1<br>ftsZ_R1	5'-ATYATGGARCATATAAARGATAG-3'<br>5'-TCRAGYAATGGATTRGATAT-3'	94°C for 2 min.<br>37 cycles: 94°C for 30 sec., 54°C for 45 sec. and 72°C for 90 sec.<br>final extension at 72°C for 10 min.<br>hold at 12°C	
fbpA	fbpA_F1<br>fbpA_R1	5'-GCTGCTCCRCTTGGYWTGAT-3'<br>5'-CCRCCAGARAAAAYYACTATTC-3'	94°C for 2 min.<br>37 cycles: 94°C for 30 sec., 59°C for 45 sec. and 72°C for 90 sec.<br>final extension at 72°C for 10 min.<br>hold at 12°C	
wsp	wsp_81F<br>wsp_691R	5'-TGGTCCAATAAGTGATGAAGAAAC-3'<br>5'-AAAAATTAAACGCTACTCCA-3'	94°C for 2 min.<br>37 cycles: 94°C for 30 sec., 52°C for 45 sec. and 72°C for 90 sec.<br>final extension at 72°C for 10 min.<br>hold at 12°C	Shoemaker et al., 2000.
Wolbachia (*w* Agra)				
w Agra_SNP01, *w* Agra *RluA* -like gene	wAgra_s2786_RluA_F<br>wAgra_s2786_RluA_R	5'-'TGGTGCATCTCGTTCAACCT-3'<br>5'-CTGCAACTAAGGCGTTCAAGG-3'	98°C for 3 min.<br>35 cycles: 98°C for 10 sec., 59°C for 30 sec. and 72°C for 100 sec.<br>final extension at 72°C for 5 min.<br>hold at 12°C	
[illegible]	wAgra_s12890_3951_F	5'-ATTTTGGCTGGCAAACTCCG-3'	98°C for 3 min.	

*w*Agra_SNP02, *w*Agra Hypothetical gene (HP_12890)	wAgra_s12890_4931_R	5'-CCTGGCTCTCTCCATCAAGC-3'	35 cycles: 98°C for 10 sec., 59°C for 30 sec. and 72°C for 100 sec.<br>final extension at 72°C for 5 min.<br>hold at 12°C	
*w*Agra_SNP3, *w*Agra Hypothetical gene (HP_2912)	wAgra_s2912_3037_F<br>wAgra_s2912_3919_R	5'-AGATATTGTAGAGGCTCTAGAGGA-3'<br>5'-TCGGTTTTGTACACTCTGCT-3'	98°C for 3 min.<br>35 cycles: 98°C for 10 sec., 54°C for 30 sec. and 72°C for 60 sec.<br>final extension at 72°C for 5 min.<br>hold at 12°C	
*w*Agra_SNP04	wAgra_s1839F<br>wAgra_s1839R	5'-AGGGTTCTCATGGGGTAGGT-3'<br>5'-TGGCCATTACAATGCCACCA-3'	98°C for 3 min.<br>35 cycles: 98°C for 10 sec., 58°C for 30 sec. and 72°C for 60 sec.<br>final extension at 72°C for 5 min.<br>hold at 12°C	
*w*Agra_SNP05	wAgra_s2235F<br>wAgra_s2235R	5'-AAGCAGAAAGTGGCTACGGT-3'<br>5'-AAGCAATGTGGTGTAAGGCA-3'	98°C for 3 min.<br>35 cycles: 98°C for 10 sec., 58°C for 30 sec. and 72°C for 60 sec.<br>final extension at 72°C for 5 min.<br>hold at 12°C	This study
*w*Agra_SNP06	wAgra_s2592F<br>Agra_s2592R	5'-AAGAGGCGATGAAGGACGTG-3'<br>5'-GTGCTCTACCCCCAACAACA-3'	98°C for 3 min.<br>35 cycles: 98°C for 10 sec., 58°C for 30 sec. and 72°C for 60 sec.<br>final extension at 72°C for 5 min.<br>hold at 12°C	
*w*Agra_SNP07	wAgra_s5121F<br>wAgra_s5121R	5'-TGAGCCGAGTGAACGAGATG-3'<br>5'-GTGTCTAGGTGCTTCTTCCCC-3'	98°C for 3 min.<br>35 cycles: 98°C for 10 sec., 58°C for 30 sec. and 72°C for 60 sec.<br>final extension at 72°C for 5 min.<br>hold at 12°C	
*w*Agra_SNP08	wAgra_s5891F<br>wAgra_s5891R	5'-ACAGCGACTAGTCCAGAGCT-3'<br>5'-CCGAGCTTTGCTTTTGTCCC-3'	98°C for 3 min.<br>35 cycles: 98°C for 10 sec., 58°C for 30 sec. and 72°C for 60 sec.<br>final extension at 72°C for 5 min.<br>hold at 12°C	
*w*Agra_SNP09	wAgra_s924F<br>wAgra_s924R	5'-TGCACCTAGAACATCACGCT-3'<br>5'-TCCCGAGTATGCACCTGAGT-3'	98°C for 3 min.<br>35 cycles: 98°C for 10 sec., 58°C for 30 sec. and 72°C for 60 sec.<br>final extension at 72°C for 5 min.<br>hold at 12°C	
*w*Agra_SNP10	wAgra_s965F<br>wAgra_s965R	5'-AGCTTGTTGTTGGCACAGTTG-3'<br>5'-TAAAGGCAACGGTCAGGGTG-3'	98°C for 3 min.<br>35 cycles: 98°C for 10 sec., 58°C for 30 sec. and 72°C for 60 sec.<br>final extension at 72°C for 5 min.<br>hold at 12°C	
